# A Time-Bound Clinical Framework for Silver Diamine Fluoride as Interim Stabilization in Severe Early Childhood Caries: Bridging Preservation to Precision with Equity and Accountability

**DOI:** 10.3390/children13060834

**Published:** 2026-06-20

**Authors:** Ziad D. Baghdadi

**Affiliations:** 1 TopSmiles Pediatric Dentistry & Orthodontics, 246 St. Anne’s Road, Winnipeg, MB R2M 3A4, Canada; drziadeddin.albaghdadi@gmail.com or zalbaghdadi@atsu.edu; 2 Butterfly Dental Group, Winnipeg, MB R3Y 1P5, Canada; 3 Children’s Dental Centre, 1630 Ness Ave, Winnipeg, MB R3J 3X1, Canada

**Keywords:** silver diamine fluoride, severe early childhood caries, interim stabilization, health equity, clinical framework, pediatric dentistry

## Abstract

**Purpose**: To provide an evidence-calibrated, time-bound clinical framework for using 38% silver diamine fluoride (SDF) as interim stabilization for severe early childhood caries (SECC) in young children, addressing gaps in existing guidelines regarding treatment duration, exit criteria, equity, and system accountability. **Methods**: This framework was developed from the American Academy of Pediatric Dentistry (AAPD) guidance (2017–2025), the 2024 Cochrane review, real-world utilization studies, and a narrative review proposing a preservation-to-precision heuristic. Recommendations are expressed using GRADE terminology. **Results**: The framework includes ten recommendations, a systems drift principle, explicit time thresholds (<6 months, 6–12 months, >12 months), a 12-month reassessment mandate, equity guardrails, a bridge vs. destination consent model, and a future research agenda. A clinical vignette contrasts appropriate short-term bridging with prolonged temporization due to access barriers. **Conclusions**: SDF is conditionally recommended for caries arrest in primary teeth. In children with SECC, SDF should be used within a documented, time-bound preservation-to-precision pathway. SDF should not become an open-ended substitute for definitive restorative care. Explicit equity implementation prevents the framework from penalizing underserved children.

## 1. Purpose

This proposed framework provides evidence-based guidance on the use of 38% silver diamine fluoride to manage severe early childhood caries (SECC) in young children. It supports SDF as an evidence-based, minimally invasive option for caries arrest and interim stabilization, while emphasizing that SDF should be used within a documented, time-bound caries management plan and should not serve as an open-ended substitute for definitive restorative care when such care is indicated [1].

The AAPD’s existing evidence-based clinical practice guideline issued a conditional recommendation, based on low-quality evidence, to use 38% SDF to arrest cavitated carious lesions in primary teeth as part of a comprehensive caries management program [1]. This proposed framework builds on that position by adding implementation language specific to young children with SECC, particularly regarding recall, reapplication, time thresholds, exit criteria, and equity considerations.

## 2. Methods

This proposed framework was developed based on:

1. AAPD evidence-based guidance and chairside resources on SDF (2017–2025) [1].
2. An evidence-calibrated viewpoint on SDF implementation [2].
3. A narrative review proposing a preservation-to-precision framework for SECC [3].
4. The 2024 Cochrane review on SDF for caries arrest (Worthington et al.) [4].
5. Real-world utilization studies [5,6,7,8].

Evidence collection procedure: A systematic search of PubMed, Scopus, and gray literature (including professional organization websites) was conducted for English-language publications from January 2017 to April 2026 using the search terms “silver diamine fluoride,” “SDF,” “early childhood caries,” “interim stabilization,” “health equity,” and “pediatric dentistry.” The references of included articles were hand-searched.

Synthesis approach: A narrative synthesis was conducted, with evidence extracted into structured tables mapping each evidence source to specific framework components (indications, time thresholds, equity considerations, etc.). No formal meta-analysis was performed.

Consensus statement: No formal Delphi panel or structured consensus process was used. This framework reflects the author’s expert synthesis of available evidence and implementation principles. Recommendations are explicitly labeled as “conditional,” “good practice statement,” or “strong good practice statement” to distinguish between evidence-based and expert-based elements.

Handling of disagreements: When evidence sources conflicted (e.g., optimal reapplication interval, comparative effectiveness of different recall schedules), the framework adopted the most conservative position consistent with the highest-quality evidence (the 2024 Cochrane review) and explicitly acknowledged the remaining uncertainty.

Development process: The framework was developed in the following steps: (1) problem identification (indefinite SDF use as a substitute for definitive care), (2) evidence review and gap analysis, (3) drafting candidate recommendations, (4) health equity impact assessment, and (5) revision for clarity and implementation feasibility.

Recommendation strength and certainty: Recommendations are expressed using GRADE terminology where applicable. Strength is categorized as strong, conditional, or a good practice statement. Certainty of evidence is categorized as high, moderate, low, or very low [4]. Strong recommendations are reserved for interventions where the balance of benefits and harms is clear despite low-certainty evidence (e.g., escalation for pain or infection). Conditional recommendations reflect uncertainty in the balance of benefits and harms. Good practice statements represent actions that are clinically obvious or ethically necessary despite the absence of direct evidence. The 12-month reassessment mandate is classified as a “strong good practice statement”—not an evidence-derived cutoff—because it reflects expert consensus on a pragmatic monitoring point. This framework does not constitute a new systematic review or formal AAPD policy; it is a structured, publication-ready clinical framework intended to align decision-making with the current certainty of evidence.

## 3. Background

### 3.1. Historical Context of SDF

Silver diamine fluoride was first described in Japan in the 1970s as a desensitizing agent for dentin hypersensitivity. Its ability to arrest caries was later recognized, leading to regulatory approval for caries management in several countries. Over the past two decades, the use of SDF has expanded considerably in pediatric dentistry (for children with early childhood caries or special health care needs) and in geriatric and public health dentistry (for frail elders or adults with limited access to restorative care). This expansion has been driven by the agent’s low cost, ease of application, and avoidance of anesthesia or drilling. However, the extension of SDF from a short-term desensitizer to a long-term caries management strategy has occurred without corresponding evidence defining the optimal duration of use or exit criteria—a gap this framework addresses.

### 3.2. Indications and Contraindications for SDF Use

Based on the AAPD guideline [1] and the 2024 Cochrane review [4], Table 1 summarizes the indications and contraindications for using 38% SDF in children with SECC.

### 3.3. Rationale for the 12-Month Reassessment Threshold

The 12-month cutoff is not derived from direct comparative evidence (which does not currently exist). Instead, it is a pragmatic, expert-based threshold informed by three converging considerations:

1. Real-world data on durability: Schlotz et al. (2024) [5] found that many SDF-treated primary teeth required additional intervention within about 2 years; a 12-month midpoint reassessment enables timely detection of treatment failure or disease progression before adverse outcomes occur.
2. Published waitlist times: Meyer and colleagues have documented that waiting times for sedation or general anesthesia in under-resourced settings often range from 6 to 12 months [6,7]. A 12-month threshold aligns with the upper end of typical system delays, after which continued SDF-only management without documented progress is difficult to justify.
3. Practicality of quality measurement: Annual time points are standard in health system quality reporting and are feasible for audit and accountability.

Alternative thresholds (6 months, 18 months, 24 months) were considered and rejected: 6 months is too short for meaningful system navigation or caregiver follow-through; 18–24 months risks exceeding the window when many teeth remain restorable without more complex interventions. The 12-month threshold is therefore a strong good practice statement based on consensus and pragmatism, not an evidence-derived cutoff. Future research should prospectively validate the optimal time thresholds.

### 3.4. Preservation-to-Precision Heuristic

For young children with SECC, SDF should be viewed as part of a preservation-to-precision pathway: a strategy to stabilize disease, buy time, and plan appropriate care, rather than a default long-term endpoint [3]. The framework proposes explicit time thresholds (<6 months, 6–12 months, >12 months) to distinguish appropriate interim stabilization from prolonged temporization [3].

Real-world data indicate that:

- Many SDF-treated primary teeth require additional intervention within about 2 years [5].
- Any delay in sedation or general anesthesia is typically measured in weeks or months, not years [6].
- Even with optimized protocols, 35% of children with caries escalate to higher-intensity treatment, and severe subtypes (Class III and V) show escalation rates of 42–50% [7].

### 3.5. Clinical Vignette: Bridge vs. Drift

#### 3.5.1. Case A—Well-Resourced, Timely Pathway

Maya, age 3.5 years, presents with SECC involving four maxillary incisors (Class III/IV). Her parents have private insurance, flexible schedules, and live near a pediatric dental clinic that offers in-office sedation. The dentist applies 38% SDF as interim stabilization, explaining that it will arrest lesions for about 8–12 weeks while a definitive treatment appointment is scheduled. Within 6 weeks, Maya receives stainless steel crowns and composites. Total time from SDF to definitive care: 2 months. SDF served as a true bridge.

#### 3.5.2. Case B—Under-Resourced, Prolonged Temporization

Elijah, age 3.5 years, has identical SECC. His family relies on Medicaid, lives 90 miles from the nearest pediatric dentist, and faces a 14-month wait for sedation at the sole public hospital that accepts their insurance. SDF is applied with a plan to “reapply every 6 months pending sedation.” Over 2 years, SDF has been applied four times. No definitive care occurs. Caregivers, never informed that SDF alone is not definitive treatment, assume the black-stained teeth are “fixed.” At age 5.5, Elijah presents with a draining fistula from a pulpal infection, requiring extraction under general anesthesia. Total time from first SDF to any definitive intervention: >24 months, with significant morbidity. SDF became a destination due to system failure.

**Why exit criteria are an equity tool**—A rigid 12-month cutoff would penalize Elijah. Instead, the framework mandates documented justification, active case management, and escalation to system accountability rather than blame.

## 4. Definitions

Table 2 outlines the principal terms used throughout this framework and provides their corresponding definitions. 

## 5. Policy Statement and Systems Drift Principle

The proposed framework recognizes 38% SDF as an evidence-supported option for arresting cavitated carious lesions in primary teeth [1,4]. Its use in young children with SECC should be integrated into a comprehensive, documented, and time-bound caries management plan that includes follow-up, risk reassessment, tooth-level triage, caregiver communication, and explicit escalation criteria.

SDF should not be presented as a universal long-term substitute for definitive restorative care in children with SECC [3,8]. When definitive care is indicated but not immediately feasible, SDF may be used for interim stabilization while an active pathway to appropriate care is pursued.

## 6. Systems Drift Principle

Definition: Systems drift is the gradual, often unintentional shift from time-limited interim stabilization to de facto long-term management, driven by the absence of accessible definitive care pathways and by a lack of corresponding clinical documentation or escalation planning. In settings with limited access to sedation, general anesthesia, or timely restorative care, SDF may be reapplied repeatedly not because it is clinically optimal, but because no realistic pathway to definitive care exists [6,8]. Current guidelines assume a functioning access system and do not address system failure [1].

Measurement: Systems drift can be measured by the proportion of SDF-treated children with a duration > 12 months who lack (a) documented exit criteria, (b) active waitlist status for definitive care, or (c) contact with a care coordinator within the preceding 6 months.

### 6.1. Practical Examples

The distinction between appropriate stabilization and systems drift is not determined solely by the duration of silver diamine fluoride (SDF) use but by the presence or absence of active case management, documentation, and efforts to secure definitive care. The following examples illustrate how identical treatment timelines may be classified differently depending on whether the clinician maintains accountability, monitors access barriers, and actively advocates for progression toward definitive treatment.
**Scenario****Classification****Rationale**Clinic reapplies SDF every 6 months for 18 months with no documentation of waitlist status, no alternative planning, and no referral.Systems driftThe absence of active case management and documentation converts the bridge to the destination.Same duration (18 months), but with verified waitlist documentation, quarterly care coordination calls, and escalation to public health authorities at 12 months.Appropriate prolonged stabilizationSystem barriers are documented, and active advocacy is occurring.

### 6.2. Accountability Pathways (Tiered)

**Clinician level:** Document barriers, specify escalation steps, and share with family.

**Practice level:** Conduct quarterly case reviews of all > 12-month SDF patients; flag cases without exit criteria.

**System level:** Submit systems barrier reports to the state Medicaid agency, public health authority, or dental accountable care organization when documented waitlists exceed 12 months.

SDF should be used within a system that actively preserves, rather than passively abandons, the child’s right to timely definitive care. When definitive care pathways are systematically unavailable, repeated SDF application without documented, time-bound escalation planning constitutes systems drift and should trigger case review, care navigation, or referral to a higher level of system accountability—not merely continued SDF reapplication [3].

Clinicians adopting this framework should document barriers to definitive care, set a maximum drift threshold (12 months for flagging), and distinguish between clinician-driven and system-driven delays across all quality metrics [3,8].

## 7. Health Equity Impact Assessment

The proposed time-bound exit criteria carry an inherent risk: without careful implementation, they could inadvertently penalize children who face the greatest barriers to definitive care [3]. Children in underserved communities often face multi-year waitlists, transportation challenges, and provider shortages [6,8]. Therefore, this framework adopts an equity-explicit approach:

1. Thresholds trigger mandatory documentation and active case management, not an automatic cessation of SDF. The 12-month point should never result in denial of SDF reapplication or in discharge from care [3].
2. Documented justification for exceeding 12 months must distinguish between system-driven and clinician- or family-driven delays. Acceptable system-driven justifications include verified waitlists longer than 12 months, repeated insurance denials under appeal, geographic maldistribution, or public health emergencies [3,6]. Unacceptable justifications include “lost to follow-up without outreach” or “convenience.”
3. When a system-driven delay is identified, the clinician’s responsibility shifts to escalation to alternative pathways or to advocacy. Options include referral to a state access program, notification of the primary care provider, submission of a systems barrier report to public health authorities, or documentation that the standard of care cannot be met because of systemic failure [3,8].
4. Outcome measures should track equity separately. Disaggregate data by insurance type, geography, race/ethnicity, and language to ensure that exit criteria are not disproportionately applied to marginalized groups [3].
5. A safety-net modification is permitted: practices serving populations with documented access barriers exceeding 12 months may operate a prolonged stabilization pathway with heightened documentation requirements (e.g., recertification every 6 months by a second clinician) [3].

Equity statement: A child in a well-resourced system who receives SDF for 24 months without definitive care is a clinical failure. A child in an under-resourced system who receives SDF for 24 months while on a verified waiting list is a systems failure [3,6]. This framework treats these scenarios differently—the former triggers clinical remediation, whereas the latter triggers system-level advocacy.

## 8. Recommendations

### 8.1. Recommendation 1: Use of 38% SDF for Initial Stabilization

The 38% SDF may be used to arrest cavitated carious lesions in primary teeth of children with SECC, provided the teeth do not show signs or symptoms of irreversible pulpal disease, necrosis, or acute infection [1,4].

**Strength:** Conditional

Certainty: Low [4]

### 8.2. Recommendation 2: Follow-Up After Initial SDF Application

Children treated with SDF should receive follow-up to assess lesion arrest and clinical stability [1]. For SECC pathways, reassessment should occur at defined intervals, generally no later than 3–6 months, with earlier review when symptoms, lesion activity, high risk, or uncertainty about arrest is present [3].

**Strength:** Good practice statement

**Certainty:** Very low [4]

### 8.3. Recommendation 3: Reapplication of SDF

Reapplication may be considered if treated lesions do not appear arrested, caries risk remains high, or clinical monitoring indicates ongoing disease activity [1]. Reapplication at approximately 3–6-month intervals may be considered, but no single interval should be considered universally optimal [2,5].

**Strength:** Conditional

Certainty: Low [4]

### 8.4. Recommendation 4: Avoidance of Fixed Interval Claims

A fixed SDF reapplication interval, including a universal 6-month interval, should not be considered the standard of care for all children with SECC [2,3].

**Strength:** Good practice statement

Certainty: Low [4]

### 8.5. Recommendation 5: Tooth Level Precision Triage

After initial stabilization, each affected tooth should be classified into one of the following categories [3,7]:

1. Appropriate for ongoing nonrestorative management.
2. Appropriate for interim restorative care.
3. Requires timely definitive restorative care.

**Strength:** Strong good practice statement

**Certainty:** Very low [4]

### 8.6. Recommendation 6: Time Bound Care Planning with 12-Month Reassessment Mandate

When SDF is used for interim stabilization, the care plan should include explicit time thresholds [3]:
**Duration****Recommendation****<6 months**Generally consistent with interim stabilization while arranging care**6–12 months**Requires documented justification and active pursuit of definitive care when indicated**>12 months** without definitive care scheduled or achieved**Mandatory reassessment and documentation.** The treating clinician must: (a) re-evaluate clinical status, (b) document specific barriers, (c) specify steps taken to overcome each barrier, and (d) justify in writing why continued SDF-only management remains appropriate. This documentation must be shared with the family and, where feasible, with a care coordinator. If no acceptable justification exists, escalation to definitive care or referral to an alternative pathway is required within 90 days [3].

**Note on the 12-month threshold:** This is a strong good practice statement based on pragmatic consensus, not an evidence-derived cutoff. See “Rationale for the 12-Month Reassessment Threshold” in the Background section.

**Strength:** Strong good practice statement

**Certainty:** Low (for the underlying evidence that reassessment is beneficial; the specific interval is consensus-based)

### 8.7. Recommendation 7: Exit Criteria and Escalation

Escalation from interim SDF-based management to definitive care or urgent reassessment is recommended when any of the following occur [3,7,8]:

- Pain or signs of infection.
- Lesion progression or failure to arrest.
- Functional compromise affecting eating, sleep, speech, or comfort.
- Caregiver preference for definitive care.
- Inability to maintain follow-up.
- More than 12 months without definitive care when definitive care is indicated (with equity guardrails as per Health Equity Impact Assessment).

**Strength:** Strong good practice statement

Certainty: Low

### 8.8. Recommendation 8: Transition to Definitive Restorative Care

Definitive restorative care should be pursued when clinically indicated and feasible, particularly for teeth with a long expected service life, structural breakdown, functional demands, or a high risk of future failure with nonrestorative management alone [3,9].

**Strength:** Strong good practice statement

**Certainty:** Low (indirect from restorative guidelines) [9]

### 8.9. Recommendation 9: Caregiver Communication and Consent (Bridge vs. Destination)

Before applying SDF, caregivers should be informed that [1,3]:

- SDF may arrest caries but does not restore tooth form, function, or esthetics.
- The staining of arrested lesions is expected.
- Follow-up and possible reapplication are required.
- Definitive care may still be necessary.
- The absence of symptoms does not equal restoring function or preventing long-term breakdown.
- SDF is used as a bridge to future care, not as the destination of care. A bridge is temporary; completing the bridge by pursuing definitive care is an expected part of the plan [3].
- If no definitive care pathway is established and actively pursued, SDF alone does not constitute completed treatment. Caregivers should be asked to confirm their understanding (e.g., “I understand that SDF is not a permanent fix”) [3].
- A written care agreement may be offered that specifies a maximum interim period (e.g., “SDF will be used for up to 6 months while we arrange sedation”) [3].

Consent should include discussion of these points and clear escalation triggers [1,3].

**Strength:** Strong good practice statement

**Certainty:** Very low [4]

### 8.10. Recommendation 10: Ongoing Nonrestorative Management, Including Longer-Term SDF Use

Ongoing nonrestorative management (with or without continued SDF application) may be considered a definitive or longer-term approach in the following well-defined scenarios, provided that lesions remain arrested, the child is asymptomatic, function is preserved, and reliable follow-up is feasible:

1. Near-exfoliation—The primary tooth is expected to exfoliate within 6–12 months, and SDF serves as definitive nonrestorative management until exfoliation.
2. Medically complex children—The risks of sedation or general anesthesia outweigh the benefits of restorative care, so SDF is continued with careful monitoring.
3. Behaviorally uncooperative children without sedation access—Documented attempts at behavior guidance and referral for sedation/GA have failed due to system barriers, and SDF is the safest available option.
4. Caregiver refusal of sedation or general anesthesia—After an informed consent discussion of risks and benefits and a documented refusal, SDF is used as a harm-reduction strategy.
5. Very low caries risk after sustained arrest—Lesions have been arrested for ≥12 months, oral hygiene and dietary risks are well controlled, and the caregiver understands the limitations of nonrestorative management.

In all these scenarios, the framework’s documentation requirements (quarterly review, care coordination, and explicit justification) remain in effect. The presence of an appropriate indication does not waive accountability; it shifts the expected management from “escalation to definitive care” to “continued monitored nonrestorative management with documented rationale.”

Strength: Conditional (for the long-term use scenarios)/Strong good practice statement (for the documentation and monitoring requirements)

Certainty: Very low

## 9. Clarification on Recommendation Strength and Evidence Certainty

Readers should note that a “strong” or “strong good practice statement” classification does not imply high-certainty evidence. For safety-related actions (e.g., escalation for pain or infection), strong wording reflects clinical necessity despite low certainty of the evidence. For time thresholds (e.g., 12-month reassessment), strong wording reflects expert consensus that a structured monitoring point is ethically and practically necessary, not that 12 months is scientifically proven to be optimal. All strong good practice statements should be revisited as higher-quality evidence emerges.

## 10. Appropriate Indications for Longer-Term SDF Use

Although the framework emphasizes timely transition to definitive care, prolonged SDF use (beyond 12 months) is appropriate in several well-defined scenarios. These scenarios should be documented with the same rigor as exit criteria:

1. Near-exfoliation (expected exfoliation within 6–12 months): SDF can serve as definitive nonrestorative management when the tooth is expected to exfoliate before adverse outcomes are likely.
2. Medically complex children: When the risks of sedation or general anesthesia outweigh the benefits of restorative care, SDF may be continued long-term with careful monitoring.
3. Behaviorally uncooperative children without sedation access: After documented attempts at behavior guidance and referral for sedation/GA have failed due to system barriers, prolonged SDF may be the safest available option.
4. Caregiver refusal of sedation or general anesthesia: After an informed consent discussion of risks and benefits and documented refusal, SDF may be continued as a harm-reduction strategy.
5. Very low caries risk after sustained arrest: When lesions have been arrested for ≥12 months, the child’s oral hygiene and dietary risks are well controlled, and the caregiver understands the limitations, ongoing nonrestorative management may be appropriate without definitive restorative treatment.

In all these scenarios, the framework’s documentation requirements (quarterly review, care coordination, and explicit justification) remain in effect. The presence of an appropriate indication does not waive accountability; it shifts the expected management from “escalation to definitive care” to “continued monitored nonrestorative management with documented rationale.”

## 11. Clinical Pathway Language for Figure Use

**Figure 1:**
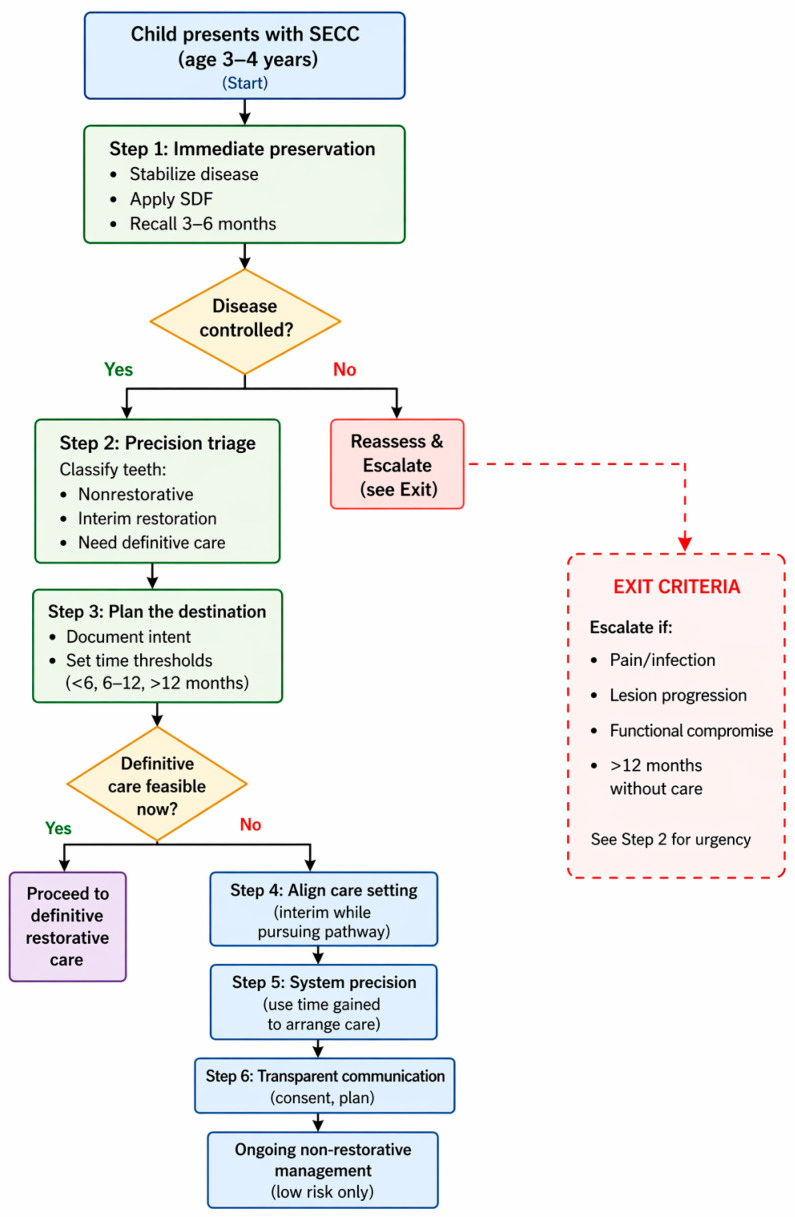
Preservation-to-precision pathway for SDF use in young children with SECC. The 12-month point is a reassessment and accountability trigger, not an automatic cessation rule.

The following steps correspond to the preservation-to-precision flowchart (Figure 1) [3].

Figure 1 Preservation-to-precision pathway for SDF use in young children with ECC. This pathway positions 38% SDF as an evidence-supported caries-arresting intervention within a comprehensive, time-bound care plan [3]. Reapplication may be considered based on lesion activity and caries risk, but no universal reapplication interval has been established [2,4]. Escalation is indicated for pain, infection, lesion progression, functional compromise, inability to maintain follow-up, or >12 months without definitive care when definitive care is indicated (with equity-explicit documentation) [3,7,8]. Real-world data indicate that many SDF-treated teeth require additional intervention within approximately 2 years, supporting planned exit criteria rather than open-ended temporization [3,5].

**Step 1.** Immediate preservation—Apply 38% SDF to indicated cavitated lesions. Establish preventive measures and schedule follow-up in 3–6 months [1,3]. Decision point: Is the disease controlled? If yes, proceed to Step 2. If not, reassess, reapply SDF, provide an interim restoration, or escalate [3].

**Step 2.** Precision triage—Classify each tooth as suitable for nonrestorative management, interim restoration, or definitive restorative care [3,7].

**Step 3.** Plan the destination—Document whether SDF is intended for interim stabilization or for longer-term nonrestorative management. Specify the monitoring interval, reapplication plan, and exit criteria using the thresholds in Recommendation 6 [3].

**Step 4.** Align the care setting—Match the treatment setting to the child’s behavioral, medical, and restorative needs [1,3].

**Step 5.** System precision—Use the time gained through SDF (typically weeks to months) to arrange definitive care, reduce barriers to access, and prevent progression [3,6].

**Step 6.** Transparent communication—Explain that SDF arrests lesions but does not guarantee durable function, esthetics, or tooth survival without additional care. Discuss exit criteria and the possibility of escalation within about 2 years [3,5].

## 12. Research and Policy Implications: Closing the Evidence–Implementation Gap

The proposed framework rests on evidence of low to very low certainty for several key implementation parameters [4]. Research is urgently needed to strengthen the evidence base and translate these principles into policy [3].

## 13. Priority Research Questions

Table 3 outlines priority research questions needed to close the current evidence–implementation gap in SDF-based care. These questions focus on treatment duration, reapplication strategies, exit criteria, equity, systems drift, and caregiver understanding, with corresponding study designs proposed to generate the evidence needed for more accountable and precise clinical decision-making.

## 14. Policy Advocacy Recommendations

1. **AAPD/ADA guideline update:** Incorporate explicit time-bound exit criteria and equity guardrails [3,10,11].
2. **State Medicaid/CHIP:** Require documented care plans and a maximum interim SDF duration (e.g., 12 months) for repeated SDF reimbursement, with exceptions for documented system barriers [3,6].
3. **Health systems and dental ACOs:** Adopt the 12-month reassessment mandate as a quality measure [3].
4. **Dental education:** Include the preservation-to-precision and systems drift principles in residency and dental school curricula [3].
5. **Research funding:** Prioritize pragmatic trials of SDF implementation strategies among underserved populations [3,4].

## 15. Comparison with Existing Guidance

Table 4 provides a structured comparison of the 2023 American Academy of Pediatric Dentistry (AAPD) policy on silver diamine fluoride (SDF) and the proposed time-bound clinical framework. The table highlights key domains—including purpose, time-bound care planning, exit criteria, equity considerations, and accountability mechanisms—and shows how the proposed framework goes beyond current guidance by operationalizing implementation. In particular, it introduces explicit duration thresholds, mandatory reassessment points, and system-level safeguards (e.g., the systems drift principle), thereby transforming SDF use from a general recommendation into a structured, auditable care pathway.

Table 5 complements this comparison by identifying major gaps in current American Dental Association (ADA) [10] guidance and mapping each gap to a corresponding solution in the proposed framework. These gaps include the absence of defined endpoints for SDF use, a lack of distinction between interim stabilization and definitive care, limited attention to equity, and insufficient accountability for prolonged temporization. The framework addresses these deficiencies through explicit exit criteria, tooth-level triage, equity-adjusted implementation strategies, and a mandated 12-month reassessment.

Together, Table 4 and Table 5 show that the framework’s primary contribution is not to challenge the evidence base supporting SDF but to translate existing evidence into actionable implementation language. By introducing time thresholds, escalation triggers, and equity-explicit safeguards, the framework closes the gap between efficacy-based recommendations and real-world care pathways, where prolonged SDF use can otherwise serve as an unintended substitute for definitive care.

## 16. Call to Action

Current evidence supports SDF as an effective caries-arresting agent but does not support indefinite, undocumented SDF use as a substitute for definitive restorative care [1,3,4]. This proposed framework offers the best available guidance—calibrated to current certainty, transparent about its limitations, and designed to evolve as higher-quality evidence emerges [3]. The author invites collaborative, multicenter efforts to validate, refine, and implement the preservation-to-precision pathway for the millions of young children worldwide affected by severe early childhood caries.

## 17. Key Summary Statement

Based on low-certainty evidence, 38% silver diamine fluoride is conditionally recommended to arrest cavitated carious lesions in primary teeth as part of comprehensive caries management [1,4]. For children with severe early childhood caries, SDF should be used within a documented, time-bound preservation-to-precision pathway that includes follow-up within 3–6 months, individualized reapplication, tooth-level triage, caregiver consent framed as a bridge rather than a destination, and explicit exit criteria with equity guardrails [3]. SDF should not be presented or used as a universal long-term substitute for definitive restorative care [3,8]. When definitive care is indicated, the time gained by SDF (typically weeks to months) should be used to pursue timely definitive treatment, not to normalize prolonged temporization as routine practice [3,6].

## Figures and Tables

**Table 1:** Indications and contraindications for 38% SDF use in severe early childhood caries.

Category	Specific Criteria
**Absolute indications**	Cavitated carious lesions in primary teeth without signs/symptoms of irreversible pulpitis or necrosis; lesions where conventional restorative care is not immediately feasible; lesions in children with behavioral or medical complexity that precludes routine restorative treatment
**Relative indications**	Deep lesions approximating the pulp but without clinical signs of irreversible disease (after careful assessment); lesions in teeth with uncertain prognosis where interim stabilization is desired pending treatment planning
**Contraindications—absolute**	Known allergy to silver or fluoride; irreversible pulpitis (spontaneous pain, tenderness to percussion, periapical radiolucency); acute infection or abscess (requires drainage and antibiotics first); non-carious tooth surface loss (no caries present)
**Contraindications—relative**	Esthetic concerns from black staining (requires informed consent); lesions that are already self-arrested; teeth expected to exfoliate within <6 months (SDF may be unnecessary); inability to achieve reasonable drying of the lesion

**Table 2:** Definitions of key terms.

Term	Definition
**Interim stabilization**	Use of SDF, preventive measures, or temporary/interim restorations to arrest or slow disease progression while monitoring the child and arranging definitive care when indicated [3].
**Definitive restorative care**	Treatment intended to restore form, function, and durability for the expected remaining service time of the primary tooth (typically >2 years for a young child), such as stainless-steel crowns, multi-surface composites with appropriate isolation, preformed crowns, or treatment under sedation/general anesthesia when clinically necessary [9].
**Disease controlled**	A clinical state in which treated lesions appear arrested (hard, darkened), the child is free from pain and infection, and oral function (eating, sleep) is maintained [3].
**Exit criteria**	Clinical or time-based triggers indicating that interim stabilization is no longer sufficient and that escalation to definitive care or urgent reassessment is needed [3,8].
**Preservation**	Conserving tooth structure when appropriate, as well as preserving the child’s comfort and emotional safety, the family’s capacity to follow through with care, and the child’s opportunity for a stable health trajectory [3].
**Precision**	Tailoring intervention to tooth-level condition, child’s overall context, and system capacity to deliver timely care, including explicit decisions on tooth-level prognosis, disease severity, follow-up feasibility, and exit criteria [3].

**Table 3:** Priority research questions to close the evidence-implementation gap.

Domain	Question	Suggested Design
Optimal duration of interim SDF	Maximum safe duration before adverse outcomes rise	Prospective cohort with time-to-event analysis [3,4]
Reapplication interval effectiveness	3-month vs. 6-month vs. as-needed schedules	Pragmatic cluster-randomized trial [2,4]
Exit criteria validation	Do explicit exit criteria improve timely definitive care and reduce adverse outcomes?	Stepped-wedge cluster randomized trial [3]
Equity impact	Do thresholds differentially affect children by insurance, rurality, and race?	Mixed methods with disparities analysis [3]
Systems drift prevalence	Among >12-month SDF cases, proportion attributable to system vs. clinician factors?	Multicenter chart audit [3,6]
Caregiver understanding	Does “bridge vs. destination” consent language improve recall and follow-through?	RCT of consent formats [3]

**Table 4:** Comparison of AAPD (2023) policy versus proposed time-bound clinical framework.

Domain	Current AAPD Guidance	Framework of this Essay	Implementation Contribution
Purpose	Supports SDF as part of an ongoing caries-management plan consistent with a dental home.	Specific to interim stabilization for SECC, with explicit time boundaries and exit criteria.	Operationalizes how to use SDF in high-burden cases, not only can it be used.
Time-bound care planning	No explicit duration limit or endpoint for stopping SDF and escalating care.	Uses thresholds of <6 months, 6–12 months, and >12 months as reassessment points.	Adds guardrails against indefinite, undocumented use of SDF.
Exit criteria	General follow-up expectations but limited operational triggers.	Defines triggers such as pain, infection, progression, functional compromise, inability to follow up, and prolonged interim care.	Prevents SDF from becoming a default long-term substitute for restorative care.
Equity impact assessment	Limited operational guidance on system-driven delays.	Includes system-driven delays, safety-net modification, and disaggregated outcome tracking.	Ensures time thresholds do not penalize underserved children.
Systems drift principle	Not explicitly addressed.	Defines drift from interim stabilization to unintended destination due to access barriers.	Recognizes a real-world failure mode ignored by efficacy-focused guidance.
Bridge vs. destination consent	Standard consent elements include staining and off-label use.	Adds explicit “bridge not destination” language and caregiver confirmation.	Transforms consent into shared accountability for follow-through.
12-month reassessment trigger	Not specified.	Requires documentation of barriers, actions taken, clinical status, and next steps.	Turns passive follow-up into auditable clinical accountability.
Tooth-level precision triage	Not operationalized for SECC implementation.	Classifies teeth into nonrestorative, interim restoration, or definitive care categories.	Moves beyond treating SECC as a single entity and supports resource allocation by tooth prognosis.
Recommendation language	Organizational guideline language.	Uses suggested implementation statements with transparent evidence, certainty, and limitations.	Avoids implying a new official guideline or consensus.
Real-world data integration	Primarily efficacy-centered.	Uses recent implementation studies on later care, delays, and escalation.	Grounds the framework in clinical practice patterns, not only controlled trials.
Clinical vignette	Not applicable.	Contrasts well-resourced bridging with under-resourced drift.	Makes equity implications concrete and clinically recognizable.
Research agenda	Calls generally for more evidence.	Maps implementation gaps to study designs.	Provides a roadmap to improve certainty and validate the pathway.

Based on AAPD policy [11] and the proposed framework [3].

**Table 5:** Gaps in current ADA guidance and corresponding solutions in the proposed framework.

Gap	ADA Public Guidance Position	Framework of this Essay
No endpoint for SDF use	Biannual applications are recommended for sustained benefit, but an endpoint is not operationalized.	Adds explicit exit criteria, time thresholds, and a 12-month reassessment trigger.
Limited distinction between interim stabilization and definitive care	Notes that restoration may be needed but does not provide a full operational pathway.	Distinguishes bridge, prolonged stabilization, selected longer-term nonrestorative management, and transition to definitive care.
Limited equity-specific implementation guidance	Assumes diagnosis, monitoring, and follow-up but does not operationalize access barriers.	Adds equity impact assessment, safety-net modification, and disaggregated outcome measures.
Limited accountability for prolonged SDF-only care	Does not define what should occur when repeated SDF continues without definitive care.	Adds documentation requirements, case review, care navigation, and system-level accountability.
Risk of rigid reapplication intervals	Biannual application is emphasized for sustained benefit.	Individualizes interval selection and cautions against treating any single interval as universal.
Limited implementation science framing	Provides evidence summary and basic protocols.	Adds systems drift principle, clinical pathway, consent tools, and research priorities.

Based on ADA policy [10] and the proposed framework [3].

## Data Availability

No new data were created or analyzed in this study.
